# Investigating sustainability in work after participating in a welfare-to-work initiative using a 2-year cohort study of Work Programme participants in Scotland

**DOI:** 10.1136/bmjopen-2023-072943

**Published:** 2024-07-03

**Authors:** Judith Brown, Simon Harold Walker, Ronald W McQuaid, Srinivasa Vittal Katikireddi, Alastair H Leyland, John Frank, Daniel Mackay, Ewan Macdonald

**Affiliations:** 1 University of Glasgow, Glasgow, UK; 2 Healthy Working Lives Group, University of Glasgow, Glasgow, UK; 3 University of Stirling, Stirling, UK; 4 MRC/CSO Social & Public Health Sciences Unit, University of Glasgow, Glasgow, UK; 5 MRC/CSO Social and Public Health Sciences Unit, University of Glasgow College of Medical Veterinary and Life Sciences, Glasgow, UK; 6 University of Edinburgh, Edinburgh, UK

**Keywords:** occupational & industrial medicine, public health, aged, health economics, health workforce, aging

## Abstract

**Abstract:**

**Objectives:**

This study investigated sustainability and multimorbidity alongside barriers to employment including health and policy to demonstrate intersectional impact on return-to-work success within a UK welfare-to-work programme.

**Design:**

Cohort study design: The study calculated the proportion of time spent employed after experiencing a job start and the proportion retaining work over 6 months. Employment/unemployment periods were calculated, sequence-index plots were produced and visualisations were explored by benefit type and age.

**Setting:**

This study used confidential access to deidentified data from unemployed Work Programme clients operated by Ingeus on behalf of the UK Government in Scotland between 1 April 2013 and 31 July 2014.

**Participants:**

13 318 unemployed clients aged 18–64 years were randomly allocated to a Work Programme provider and monitored over 2 years.

**Results:**

This study has two distinct groupings. ‘Employment and Support Allowance (ESA)’ corresponding to those with work-limiting disability in receipt of related state financial support, and ‘Jobseeker’s Allowance (JSA)’ corresponding to unemployment claimants. Despite fewer and later job starts for ESA clients, those that gained employment spend relatively more subsequent time in employment when compared with individuals without work-limiting conditions (ESA clients under 50, 0.73; ESA clients over 50, 0.79; JSA clients under 50, 0.67 and JSA clients over 50, 0.68). Proportion in permanent jobs was higher among ESA than JSA clients (JSA under 50, 92%; JSA over 50, 92%; ESA under 50, 95% and ESA over 50, 97%).

**Conclusion:**

The research demonstrated that returning to paid employment after a reliance on welfare benefits is challenging for people aged over 50 and those with disability. The study found that although fewer older ESA claimants entered employment, they typically remained in employment more than JSA clients who did not leave the Work Programme early. This indicates the importance of identifying risk factors for job loss in ageing workers and the development of interventions for extension of working lives.

**STRENGTHS AND LIMITATIONS OF THIS STUDY**

- The 2-year longitudinal study allowed consideration of a range of potential employment-related outcomes for participants and how they changed over time.
- In addition to descriptive statistics, sequence analysis allowed visualisation of participant trajectories over the period.
- The main employment data were used for payments by government and are considered to be reliable.
- There is no information on experiences after the 2-year follow-up.
- Jobseeker’s Allowance clients who left the programme early (often due to getting long-term employment) are excluded from some of the analysis.

## Introduction

 Historically, job seekers with disabilities and chronic illnesses experience high rates of unemployment and low rates of re-entering employment.^1^ This is a particular problem in the UK, which currently has the highest proportion of people on disability benefits in the Organisation for Economic Co-operation and Development.^2^ This rate is expected to rise considering the ageing demographic of the UK workforce, evidenced by employment rates for the working age population declining sharply from over 80% of 50 years in the UK, to around 60% of 60 years with a steeper decline at older ages.^3^ UK government policy remains dedicated to narrowing the disability employment gap with the targeted return of one million people into work with welfare-to-work programmes being expanded to include more of those out of work due to chronic illness or disability.^4^ The successors to the government’s Work Programme in 2017 are more focused on unemployed job seekers with health issues, with both the Work and Health Programme in England and Fair Start Scotland specifically targeting those with disabilities, health conditions and vulnerable people such as the long-term unemployed.^5^ This research study considered the experiences of a cohort of 13 318 unemployed clients with a range of ages randomly allocated to a Work Programme provider for the duration of 2 years. While an earlier study conducted by the Healthy Working Lives Group (2018) identified that age, the number of health conditions, multimorbidity, duration of unemployment, deprivation and educational level all impacted the likelihood of return to work, it was clear that evidence related to the impact of advanced age, chronic illness or disabilities was still lacking and demanded further consideration in relation to the acquisition and sustainment of employment within the UK, and particularly for the focus of this study, Scotland.^6^

In the UK, of the 3.7 million disabled people out of work in 2016, about 10% per year (400 000) moved into work compared with around 30% for non-disabled people. Roughly consistent figures were found for people with long-term mental health problems by the Stevenson and Farmer review of mental health and employers.^7^ The employment rate gap between those with and without disabilities rose during COVID-19 but returned to near prepandemic levels by 2022, although overall employment rates fell due partly to increased long-term illness. According to the 2022 Scottish Government report, Scotland has the lowest life expectancy of the nations within the UK.^8^ The Scotland Health and Demographic profile indicated that Scotland has continually faced significant issues with multimorbidity and long-term ill health which has a wider impact on work and opportunities to remain in work.^7^

Crucially, the Scottish-focused Work Programme differed from previous programmes by focusing results on clients sustaining, rather than just entering, employment and paying providers almost entirely by results (ie, an outcomes-based approach).^9^ Providers claimed a job outcome payment after a client had moved into and been in employment for 3 or 6 months, depending on how far they were from the labour market (ie, which payment group they were in). After receiving an initial job outcome payment, providers claimed sustainment payments for every 4 weeks a client remained in employment, again depending on the client’s payment group (either 13, 20 or, for those furthest from the labour market, 26 four-weekly payments). The payments were not for a particular job but for accumulated employment, irrespective of how many employers or jobs contributed to this time in employment. Many other studies have reported employment outcomes based on job outcome payments and sustainment payments rather than amount of time in individual jobs.^10^ This study aimed to add to the literature by clarifying the characteristics of in and out-of-work experiences of work programme clients after their initial return to work and consider their longevity of employment.

## Methods

In this study, through co-operation with Ingeus, a contractor to the department of work and pensions (DWP), serving all of Scotland, we considered the range of jobs (including start and end dates) undertaken by 13 318 clients enabling us to investigate the characteristics of each client, job and transitions between unemployment and employment. By conducting a detailed analysis of a cohort of Ingeus clients, including calculation of employment and unemployment periods, cross-examined with sequence-index plots and visualisations by benefit type, age and health conditions, this study contributes to new understandings of the sustainability of employment.^11^

### Study design and data collection

The research team was granted confidential access to deidentified data from unemployed clients aged 18–64 years who entered the Work Programme operated by Ingeus on behalf of the UK Government in Scotland between 1 April 2013 and the 31 July 2014. All participants were randomly allocated by the Job Centre/DWP to one of two private providers (including Ingeus). Cohort members were then followed up longitudinally for the 2 years after they were first engaged in the Work Programme. Participants are classified as ‘clients’ for the rest of this paper, in keeping with the vocabulary of Work Programme. Clients were divided into four groups: Job Seekers Allowance (JSA) (normally awarded if no health conditions declared) clients aged under 50 referred to as ‘JSA under 50’, JSA clients aged 50 and above (‘JSA over 50’), Employment and Support Allowance (ESA) (normally awarded if health conditions declared)clients aged under 50 (‘ESA under 50’) and ESA clients aged 50 and over (‘ESA over 50’).^6^ Each client was individually assessed to track their employment experience and sustainment over the course of the study. Each client was also categorised by key variables including age (below or above 50), gender, highest level of qualification, ethnicity, caring responsibilities and Scottish Index of Multiple Deprivation classification (online supplemental file 1).

Each job undertaken by the client was determined and classified according to the Standard Occupational Classification 2010 (SOC) major groups^12^ and the number of hours worked. Full-time work was defined as 30+ hours and part-time work was defined as under 30 hours. The Labour Force Survey^13^ uses respondents’ self-defined full-time work classification with a median of around 34 hours per week for women working full time over the study period so if 35+ hours was taken as full-time work then over half of these women would be excluded. Results were tabulated and associations across the four client groups by occupation type and hours worked analysed using χ^2^ tests. Clients were asked about their health by their employment advisor at the start of their engagement with the Work Programme and all health conditions disclosed were recorded. The research team recoded the health conditions into seventeen categories (including ‘no health conditions’). Clients disclosed between one and seven health conditions (online supplemental table 2).

Sequence analysis has been used to model careers and life-course analysis in work and household interactions.^14^ In this study, the outcome variable was the client’s employment status during their 2-year engagement with the Work Programme. Sequence analysis was used to explore the unemployment-to-employment (and employment-to-unemployment) transitions made by clients.^15^ For those clients who started a job, each unemployment and employment spell during their 2-year period in the Work Programme was determined and sequence index plots were produced using Stata V.14 for the four client groups. Three different categories of labour market participation during the 2 year follow-up period were possible: (1) unemployment, (2) employment or (3) left Work Programme. In this study, 1187 clients left the programme before 2 years, as they entered employment quickly and remained in work and no further information was available on them.

The horizontal axis represents the clients’ time in the Work Programme from day 1 at the left to day 727 (ie, 2 years) at the right. Each point shows a specific category at a particular time point while the categories themselves are reflected by a different colour (unemployed—yellow, employed—blue and left programme—red). The three dimensions of sequence data (observations, time points and categories) are plotted in one single graph. Each line on the vertical axis represents an individual client. However, with an increasing number of individual clients, the lines that have to be displayed in the graph become thinner and overplotting results.^16^ For clarity, the sequence index plots shown in this paper are a random subsample of 100 clients and were ordered by time to first job start. The consistency of results with other random subsamples was checked and led to no substantive differences in interpretation.

Previous studies have used a volatility indicator and an integration indicator as sequence analysis assessment measures.^17 18^ In this study, we have derived two values appropriate to the data to interpret the sequence plots—a Time in Employment Index (TIEI) and a Work Retention Index (WRI). The TIEI is the proportion of total time spent in employment while engaging in the Work Programme (either the full 2 years or time before leaving early, as described above for 1187 clients). These latter clients (13% of all JSA clients or 21% of those getting a job) are likely to have remained in employment after leaving the programme but cannot be assumed to have done so, hence these results reflect JSA clients not leaving the programme early (ie, those who generally are expected to have required considerable support), rather than all JSA clients. These results are presented as mean and median values. As these are similar, analysis of variance and Bonferroni correction were used to test differences across the four groups. The WRI reflects how well clients sustained employment after their first job start on the programme.

Additionally, a 6WRI (6-month WRI) was developed to identify if the censoring of the data influenced the results recorded (ie, some ESA clients started work near the end of their 2 years on the WP and we did not have client data after that time). This index, therefore, only considered employment during the 6 months after first starting a job and excluded all those who first started a job in the last 6 months of the Work Programme (n=614). For each of the four groups of clients, this was measured as the proportion of time in employment after their first job start and was determined as follows: the total number of days worked by all clients as a proportion of the maximum number of days possible between the day of first job start to the final day of programme, that is, day 727 (or exit from programme). P values were obtained using proportion tests. Although the sequence plots show only a random sample of 100, data from all clients were included in the TIEI and WRI calculations.

### Patient and public involvement

The research conducted was carried out on quantitative data obtained through the DWP in conjunction with Ingeus who at the time of the data collection acted as data processor on behalf of the data controller, the DWP. All of the outgoing findings have continued to be shared with the DWP, and publications resulting in this research have been made available to the DWP to share. As the individual participant was anonymised through the process of codification, it is impossible to share the data directly with each individual participant. Patients were not involved within the direct creation or carrying out of the research study.

## Results

### Job start by benefit type and age group

6479 of the total 13 318 clients (49%) started at least one job during their 2-year engagement with the Work Programme and the total jobs started were 10 696 over their time in the Work Programme (ie, 1.65 jobs per client). Job start rates were ‘JSA clients under-50’, 65%; ‘JSA clients over-50’, 49%; ‘ESA clients under-50’, 23%; ’ESA clients over-50’, 14% (online supplemental table 3). Of those starting a job, JSA clients had a greater likelihood of starting more than 1 job than ESA clients (JSA under 50, 43%; JSA over 50, 39%; ESA under 50, 29% and ESA over 50, 24%).

### Job start by benefit type and gender

4204 male clients of the total 13 318 clients (31%) started at least one job during their 2-year engagement with the Work Programme while 2275 female clients (17%) also started at least one job with the Work Programme. 61% of the cohort were male (8059 clients) with 52% starting at least one job over the course of the programme. The female clients (5259) represented 39% of the overall cohort, with 43% of the female clients starting at least on job during their 2-year engagement. A strong negative relationship between age and having a job start for both JSA and ESA clients is visible within the findings. As shown, male JSA clients have a higher probability of job start compared with female JSA clients of all ages. This has been considered in further detail within Neary *et al*, ‘Age Health and other Factors’.^19^

Total jobs started were 10 696 over their time in the Work Programme (ie, 1.65 jobs per client). Job start rates were ‘JSA clients under-50’, 65%; ‘JSA clients over-50’, 49%; ‘ESA clients under-50’, 23%; ’ESA clients over-50’, 14% (online supplemental table 4). Of those starting a job, JSA clients had a greater likelihood of starting more than 1 job than ESA clients (JSA under 50, 43%; JSA over 50, 39%; ESA under 50, 29% and ESA over 50, 24%).

### Characteristics of jobs started by benefit type and age group

**Figure 1:**
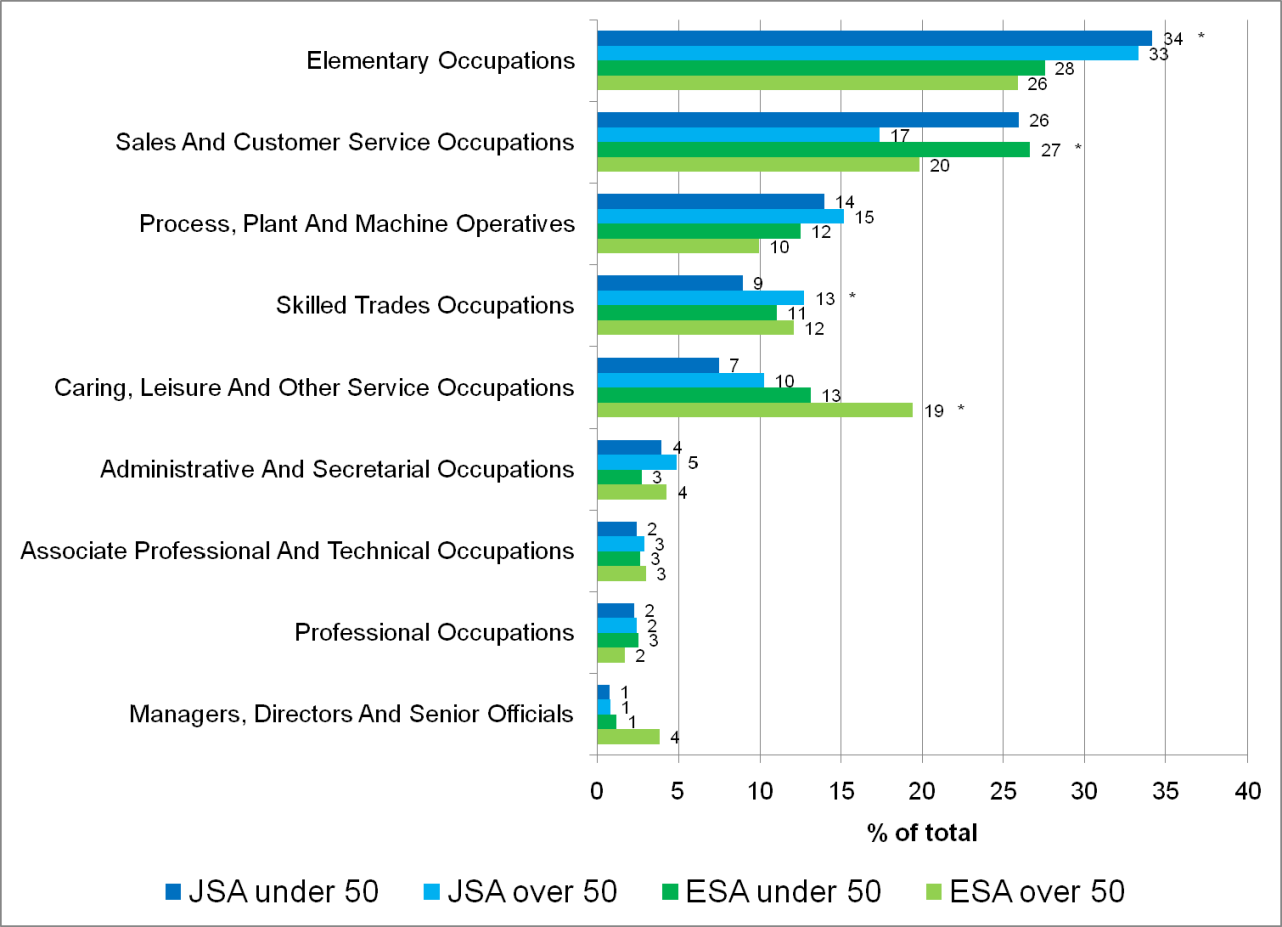
Type of jobs started (SOC code) by benefit type and age group (expressed as percent of each client group). *Test of association on each SOC and four client groups p values <0.001. ESA, Employment and Support Allowance; JSA, Jobseeker’s Allowance; SOC, Standard Occupational Classification.

JSA clients were more likely to undertake elementary occupations than ESA clients (figure 1). Younger clients (both JSA and ESA) were more likely to undertake sales and customer service occupations than older clients. Younger JSA clients were less likely to undertake skilled trades occupations, partly reflecting their lower work experience. ESA clients were more likely to undertake caring, leisure and other service occupations. Approximately 63% of jobs started by JSA clients (both age groups) were full-time (JSA under 50, 62.5%; 63.2% JSA over 50s). ESA clients, especially older clients, undertook less full-time work (ESA under 50, 51.5%; ESA over 50s, 41.8%).

### Health conditions disclosed by benefit type and age group

In terms of the self-reported health of clients, online supplemental table 2 sets out the percentage of each of the four groups that disclosed specific health conditions. 81% of JSA claimants under 50 and 58.6% of JSA claimants over 50 declared no health conditions (a total of 78% of JSA claimants combined) and 4.8% and 3.3% of younger and older ESA participants reported no health conditions. While a disparity is to be expected, the percentages potentially reflect response errors or participants’ perception of their own disability as not being significant. Evidence for the inability, or unwillingness, of individuals to recognise or self-identify as experiencing ill health conditions was described by Goering.^20^

Statistically, three main types of condition particularly affect ESA participants (musculoskeletal, anxiety and depression). Musculoskeletal problems in ESA participants (34.9% for <50 years and 60.4% for >50 years) were more than JSA clients (5.2% and 20.9%, respectively). Large proportions of ESA clients disclosed mental health conditions: depression (younger ESA 47.3%, older ESA 50% compared with 4.3% and 6.6% for JSA respectively); anxiety (36.5% and 28.6% for younger and older ESA clients and 2.2% and 2.5% for JSA clients, respectively). Younger ESA participants had more severe mental health issues (6.9%) and also disclose more addictions (12.8%). Older JSA suffer relatively more from diabetes (5.2%).

### Sequence analysis of employment spells by benefit type and age group

**Figure 2:**
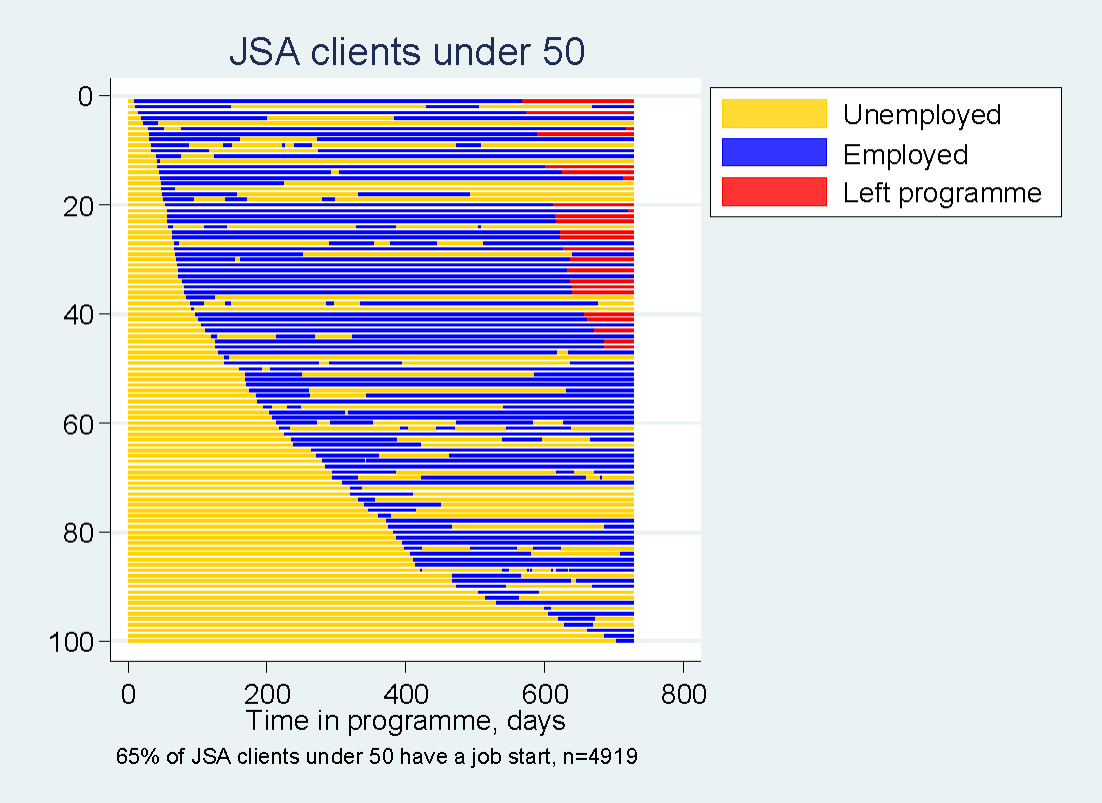
Sequence analysis showing unemployment-to-employment transitions for JSA clients under 50. JSA, Jobseeker’s Allowance.

By creating sequence index plots for 100 clients in each group for 2 years from the first day of the Work Programme (figure 2), it is possible to visually chart their individual experiences and the sequence analysis indicated both variations and similarities in outcomes between the four groups of clients. More JSA clients entered employment and they had shorter periods of unemployment prior to their first job start (online supplemental table 4). However, in terms of employment sequences, the visualisations reveal that the ESA clients spent more time in employment after the first job start, which was corroborated by the assessment measurements (online supplemental table 4). JSA clients were more likely to leave the programme early (red horizontal lines) as they remained in employment, but this finding may also have been influenced by different payment rules for providers to track past JSA and ESA clients.

### Sequence analysis assessment measures

The mean and median values of the TIEI are shown in online supplemental table 5. The mean TIEI for ESA under 50 clients was 0.40, significantly lower than the two JSA values (0.48 and 0.47), but not than the ESA over 50 value (0.45). There was no difference in the TIEI for ESA over 50 clients vs all JSA clients. The WRI (proportion of time in employment after the first job start) for the four client groups was as follows: ESA clients under 50, 0.73; ESA clients over 50, 0.79; JSA clients under 50, 0.67; JSA clients over 50, 0.68 (online supplemental table 4). All the values were statistically different from each other indicating that ESA clients spend relatively more time in employment after they start their first job and that although fewer of the over 50 ESA clients returned to work, those that did had the highest WRI of the whole study population. Results of the third index 6WRI were similar after censoring of the data.

In summary, these data show that typically younger ESA clients undertake significantly less accumulated employment during the 2-year programme (TIEI) compared with all other clients (JSA clients and older ESA clients) typically because ESA clients on average take longer to start their first job. However, once in work, ESA clients have relatively more accumulated employment compared with JSA clients; and older ESA clients had a longer period of sustained work after job start compared with the younger ESA clients (almost 10% greater) (WRI). The third index (6WRI), which considers censoring and excludes clients who got a job in the last 6 months of the programme, shows statistically significant differences across the four client groups. The average differences between the under 50 and over 50 age groups are less than 5% of the mean which suggests that these results must be taken with caution. Still, the results strongly suggest that greater support needs to be given to ESA clients who are under 50.

## Discussion

Once people lose their jobs it is much harder to get them to return to work particularly if this has been for a long period or with disabilities or other health issues.^6^ This study has demonstrated the importance of multimorbidity and extrapolated the findings of other ageing worker studies which identified risk factors for older workers falling out of work.^14^ These factors include sickness absence, hospital appointments, multimorbidity, worker expectations and lack of skills. As previously stated,^19^ older workers are generally more likely than younger workers to have multiple chronic health problems, as multimorbidity (the presence of two or more associated chronic health conditions) and disability increases with age and in this population the likelihood for returning to work for the unemployed was inversely related to the number of health conditions. These findings support the need for improved occupational healthcare of ageing workers, as well as specific health surveillance to identify modifiable risk factors of job loss.

The Work Programme aimed to support unemployed people and those with disabilities and health issues find jobs and remain in employment.^6^ This study was able to investigate trajectories of labour market participation while engaging with the Work Programme and improve understanding of multiple employment–unemployment transitions after the first job start. We had access to high-quality individual jobs data (used to report to and claim payments from the UK Government) as well as job outcome and sustainability data. Further, we were able to study four policy-relevant groups of clients. JSA clients have been described as ‘easier-to-help’ and ESA ‘harder-to-help’ and the Work Programme sought to narrow the gap in employment rates between the two groups and reduce the levels of ‘parking’ of the ‘harder-to-help’.^6^ The age category split is influenced by the classification by the UK Government of the age of 50 as being a key demarcation point for the beginning of significant issues in retaining or returning to work. This is not to say that further research is not required to focus on the differences between age groups, however, it is evident for the purposes of this research that the split at 50 has significant consequences for individuals. This has previously been outlined by the ONS (2022), most recently in association with the COVID-19 epidemic.^21^

### Sequence analysis

This is the first study, using sequence analysis, to examine the trajectories of labour market participation between four groups of clients engaging with the UK’s Work Programme. Our research shows that JSA clients returned to work more quickly and were likely to have more jobs, than ESA clients (ie, those with greater health conditions) who were much less likely to move into work, but when they did so older ESA clients consistently did better in sustaining employment longer term. The reasons for this are uncertain, but factors may be due to greater intervention support and biopsychosocial factors such as their greater work experience. This accords with the biopsychosocial model of health.^22^ The novel findings presented in this paper have important implications for policy, notably that long-term disabled ESA clients should not be written off as 8.2% of claimants participating after 11 years of unemployment returned to work as has previously been shown in follow-up studies of retirement on the grounds of ill health.^13^ The Work Programme mandated that ESA clients should be encouraged to undertake work-related activity, but unlike their JSA counterparts could not be mandated in any way to undertake work, apply for jobs or undergo medical treatment. This may partly explain the significant difference in uptake of roles between JSA and ESA clients.^22^

This study differs from prior research in that participants were considered beyond the initial confirmation of a new job leading to a more optimistic outlook than Grover and Piggott^23^ as our research highlighted that ESA participants who secured employment were no more likely to enter a ‘low pay, no-pay’ cycle than other participants. The research also demonstrated a relatively low academic achievements across the cohort with most clients likely to undertake elementary occupations. Older ESA clients were more likely to occupy caring, leisure and other services positions and all older clients (including JSA) were less likely to undertake pressured sales and service occupations. JSA clients also undertook more full-time jobs than ESA clients reflecting increased part-time working by ageing workers. Older workers who were out of work also face significant barriers in the labour market and are less likely to regain employment after job loss and are at increased risk of chronic health conditions and multimorbidity which contribute to job loss and may make re-employment difficult.^24^ In addition, this age group may encounter barriers and factors that interact with their health including direct and indirect age discrimination,^25^ skills gaps, caring responsibilities^26^ and a lack of flexible working opportunities.^27^ Therefore, investigating younger and older JSA and ESA clients are strengths of the study.

A comparative study which considered the impact of job types related to the success of welfare-to-work programmes published similar results to our research yet was limited to approximately 800 clients and lacked recognition of differing client characteristics.^28^ Previous sequence analysis studies have used volatility indicators and integration indicators. We have derived three new indicators to assist interpretation of the sequence plots and investigate all possible work outcomes. The TIEI shows ESA clients under 50 spent less time working overall in the programme which was due to longer times to start their first job. Conversely, the WRI ESA clients spent relatively more of their time after their first job start in employment.

Further strengths of the study include low rates of attrition due to the incentives for continued data collection. Similarly, the recruitment selection was random, and the generalisability of the sample and use of administration data helped address issues of non-response which have limited other analyses.

### Study limitations

While the availability of such rich data on unemployment to employment transitions is a major strength of this study, there are also some limitations to the data. Once Ingeus received all eligible payments for a client, no further data on that client were available. Hence, we created the ‘left programme’ status for these 1187 clients. Although these clients may have remained in employment due to their good work history for the remaining 2 years of the Work Programme, we could not assume this, so we censored follow-up for subjects who ‘left the programme,’ in our calculation of the TIEI and WRI. These clients were generally JSA clients (due to payment rules mainly payment groups 1 and 2, who had found work quickly and sustained in the same job. They will have had to work for at least 550 days for Ingeus to collect all payments (and record their data). The only clients who could not leave the Work Programme early were ESA clients from payment groups seven and nine because of the different payment structure with much longer job outcome and sustainment payments that providers would collect for these clients.

In terms of the characteristics of jobs, we were provided with SOC code and hours worked. The Ingeus dataset also contained information on the type of contract (permanent, fixed term or not defined). Across the four benefit groups, the data suggested that approximately 95% of the jobs started by these clients were permanent (JSA under 50, 92%; JSA over 50, 92%; ESA under 50, 95%; ESA over 50, 97%). As part of the Work Programme evaluation, Meagre *et al* found 48% of jobs were permanent or open-ended contract.^29^ Due to this large difference between the two studies, we had to question the reliability of our data in terms of contract type.

### Conclusion

The main novel findings from this study are that ESA client sometimes perform relatively better than JSA clients in terms of accumulated employment after they started their first job, has significant implications for the new welfare-to-work programmes replacing the Work Programme across the UK, and the government’s commitment to reduce the disability employment gap.^1^ However, far fewer ESA clients made the transition into employment strategies and evidently tailored interventions for clients to secure jobs are essential alongside focused work support such as job coaching to prevent role attrition.

Programmes to enhance the employability of unemployed people and help them to find and sustain work can include two main types.^30^ ‘Work First’ is a programme which focuses mainly on compulsory job search and short-term interventions to facilitate a quick return-to-work (with expected improvements in job-related skills and work experience); and human capital development programmes which tailor services to promote longer-term skills (eg, typically via education and training), personal development (where prejob entry preparation, postjob support, support with issues such as anxiety, low mood, etc) and/or careful matching between client and available jobs.^5^ Although we do not have evidence, a limited part could also be played by labour demand factors if employers sought to retain ESA clients, for instance, as part of their disability diversity policies. The results indicate that even when JSA clients start work, they experience extensive periods out of work (around a third of the time, out of work for JSA clients) and tend to work in lower skilled jobs. This suggests that it may be useful to invest in, for instance, further skills and careers development for people on such ‘work first’ programmes to increase their likelihood of remaining in employment and progress in their career (and usually enjoy the associated income and health-related benefits) rather than by entering the first available job, that is, this suggests support for a more ‘human capital development’ approach.^31^ The results also suggest that the extra time and support for those on ESA may lead to sustained benefits of more secure and probably longer-term employment. We have previously shown that the likelihood of return to work for the unemployed is inversely related to the number of health conditions.^5^ These findings would suggest that improved occupational health and clinical care of ageing workers with multiple morbidity should be evaluated to assess their ability to reduce the risk of job loss, and to this end it has been suggested that health surveillance of the older workers should be considered.

## supplementary material

10.1136/bmjopen-2023-072943 online supplemental file 1

10.1136/bmjopen-2023-072943 online supplemental file 2

10.1136/bmjopen-2023-072943 online supplemental file 3

10.1136/bmjopen-2023-072943 online supplemental file 4

10.1136/bmjopen-2023-072943 online supplemental file 5

## Data Availability

Data are available on reasonable request.
