# Supplementary material for: Investigating sustainability in work after participating in a welfare-to-work initiative using a 2-year cohort study of Work Programme participants in Scotland
Source: BMJ Open. 2024 Jul 3;14(7):e072943. doi: 10.1136/bmjopen-2023-072943 (PMC11733907; doi:10.1136/bmjopen-2023-072943)
Supplement: online supplemental file 1 [file bmjopen-14-7-s001.pdf]

# Table 1. Descriptive Characteristics and job start of the SOPIE cohort by benefit type.

| Descriptive characteristics and job start of the SOPIE cohort by benefit type | | | | |
|---|---|---|---|---|
| | JSA clients | | ESA clients | |
| | No. of clients (% of total JSA clients) | No. of clients with job start (% of each category with job start) | No. of clients (% of total ESA clients) | No. of clients with job start (% of each category with job start) |
| **Benefit type** | **8996** | **5612 (62.4%)** | **4322** | **867 (20.1%)** |
| **Individual factors** | | | | |
| Age (years) | | | | |
| <50 | 7590 (84.4%) | 4919 (64.8%) | 3000 (69.4%) | 685 (22.8%) |
| >50 | 1406 (15.6%) | 693 (49.3%) | 1322 (30.6%) | 182 (13.8%) |
| Gender | | | | |
| Male | 5799 (64.5%) | 3754 (64.7%) | 2260 (52.3%) | 450 (19.9%) |
| Female | 3197 (35.5%) | 1858 (58.1%) | 2062 (47.7%) | 417 (20.2%) |
| Length of prior unemployment | | | | |
| 0–6 months | 638 (7.1%) | 476 (74.6%) | 128 (3.0%) | 73 (57.0%) |
| 7–12 months | 2034 (22.6%) | 1498 (73.7%) | 264 (6.1%) | 118 (44.7%) |
| 1–2 years | 3510 (39.0%) | 2416 (68.3%) | 733 (17.0%) | 261 (35.6%) |
| 3–5 years | 1072 (11.9%) | 571 (53.3%) | 802 (18.6%) | 173 (21.6%) |
| 6–10 years | 886 (9.9%) | 399 (45.0%) | 885 (20.5%) | 119 (13.5%) |
| 11+ years | 856 (9.5%) | 252 (29.4%) | 1510 (34.9%) | 123 (8.2%) |
| Highest qualification | | | | |
| Degree or higher | 580 (6.5%) | 436 (75.2%) | 165 (3.8%) | 64 (38.8%) |
| A levels/NVQ level 3 and equivalent | 1443 (16.0%) | 1006 (69.7%) | 480 (11.1%) | 149 (31.0%) |
| Five or more GCSEs grades A*–C and equivalent | 1564 (17.4%) | 1094 (70.0%) | 468 (10.8%) | 134 (28.6%) |
| Under 5 GCSEs A*–C and equivalent | 2145 (23.8%) | 1317 (61.4%) | 975 (22.6%) | 185 (19.0%) |
| Below GSCE level | 3264 (36.3%) | 1759 (53.9%) | 2234 (51.7%) | 335 (15.0%) |
| Ethnicity | | | | |
| White British | 7950 (88.4%) | 4906 (61.7%) | 4062 (94.0%) | 813 (20.0%) |
| Other | 1046 (11.4%) | 706 (67.5%) | 260 (6.0%) | 54 (20.8%) |
| Have health concerns which believe will affect ability to work | | | | |
| No | 7247 (80.6%) | 4984 (68.8%) | 255 (5.9%) | 126 (49.4%) |
| Yes | 1749 (19.4%) | 628 (35.9%) | 4067 (94.1%) | 741 (18.2%) |
| Number of health conditions disclosed | | | | |
| 0 | 6365 (70.8%) | 4399 (69.1%) | | |
| 0 and 1 | | | 1290 (29.8%) | 381 (29.5%) |
| 1 | 1727 (19.2%) | 905 (52.4%) | | |
| 2 | 608 (6.8%) | 239 (39.3%) | 1396 (32.3%) | 296 (21.2%) |
| 3 | | | 896 (20.7%) | 123 (13.7%) |
| 3 or more | 296 (3.3%) | 69 (23.3%) | | |
| 4 | | | 425 (9.8%) | 40 (9.4%) |
| 5 or more | | | 315 (7.3%) | 27 (8.6%) |
| Client perception of job start—When do you see yourself starting work? | | | | |
| Within 1 month | 2188 (24.3%) | 1646 (75.2%) | 92 (2.1%) | 56 (60.9%) |
| 2–3 months | 3268 (36.3%) | 2258 (69.1%) | 248 (5.7%) | 142 (57.3%) |
| 4–6 months | 875 (9.7%) | 486 (55.5%) | 262 (6.1%) | 117 (44.7%) |
| [illegible] | [illegible] | [illegible] | [illegible] | [illegible] |
| **Personal circumstances** | | | | |
| Caring responsibility for anyone other than children | | | | |
| No | 8561 (95.2%) | 5398 (63.0%) | 4046 (93.6%) | 822 (20.3%) |
| Yes | 435 (4.8%) | 214 (49.2%) | 273 (6.4%) | 45 (16.3%) |
| Housing | | | | |
| Homeowner | 401 (4.5%) | 261 (65.1%) | 323 (7.5%) | 95 (29.4%) |
| Living with family | 3049 (33.9%) | 2155 (69.4%) | 609 (14.1%) | 146 (24.0%) |
| Rented private | 1304 (14.5%) | 850 (65.2%) | 563 (13.0%) | 126 (22.4%) |
| Rented social | 3853 (42.8%) | 2212 (57.4%) | 2655 (61.4%) | 469 (17.7%) |
| Insecure | 389 (4.3%) | 174 (44.7%) | 172 (4.0%) | 31 (18.0%) |
| Parental status | | | | |
| No children | 4892 (54.4%) | 3218 (65.8%) | 1693 (39.2%) | 329 (19.4%) |
| Children, two parent family | 622 (6.9%) | 406 (65.3%) | 288 (6.7%) | 89 (30.9%) |
| Children, shared custody/not living with you | 1254 (13.9%) | 735 (58.6%) | 711 (16.5%) | 145 (20.4%) |
| Children, lone parent family | 1369 (15.2%) | 800 (58.4%) | 597 (13.8%) | 135 (22.6%) |
| Children, adults living at home/ adults not living at home | 859 (9.6%) | 453 (52.7) | 1033 (23.9%) | 169 (16.4%) |
| External factors | | | | |
| SIMD quintiles | | | | |
| 1 (most deprived) | 4779 (53.1%) | 2899 (60.7%) | 2362 (54.7%) | 411 (17.4%) |
| 2 | 2075 (23.1%) | 1302 (62.8%) | 1013 (23.4%) | 219 (21.6%) |
| 3 | 1151 (12.8%) | 707 (61.4%) | 550 (12.7%) | 130 (23.6%) |
| 4 | 603 (6.7%) | 419 (69.5%) | 226 (5.2%) | 57 (25.2%) |
| 5 (least deprived) | 388 (4.3%) | 285 (73.5%) | 171 (4.0%) | 50 (29.2%) |
| Sixfold urban rural classification | | | | |
| 1 Large urban areas | 4573 (50.8%) | 2891 (63.2%) | 2578 (59.7%) | 467 (18.1%) |
| 2 Other urban areas | 3176 (35.3%) | 1962 (61.8%) | 1237 (28.6%) | 275 (22.2%) |
| 3 Accessible small towns | 623 (6.9%) | 395 (63.4%) | 232 (5.4%) | 57 (24.6%) |
| 4 Remote small towns | 173 (1.9%) | 87 (50.3%) | 69 (1.6%) | 20 (29.0%) |
| 5 Accessible rural | 333 (3.7%) | 211 (63.4%) | 151 (3.5%) | 38 (25.2%) |
| 6 Remote rural | 118 (1.3%) | 66 (55.9%) | 55 (1.3%) | 10 (18.2%) |

Test of association on all variables and benefit type, $p<0.001$, except for test of association on SIMD quintiles and benefit type, $p<0.05$.

ESA, Employment and Support Allowance; GCSE, General Certificate of Secondary Education; JSA, Jobseeker's Allowance; NVQ, National Vocational Qualification; SIMD, Scottish Index of Multiple Deprivation; SOPIE, Supporting Older people into Employment.
