# Supplementary material for: Investigating sustainability in work after participating in a welfare-to-work initiative using a 2-year cohort study of Work Programme participants in Scotland
Source: BMJ Open. 2024 Jul 3;14(7):e072943. doi: 10.1136/bmjopen-2023-072943 (PMC11733907; doi:10.1136/bmjopen-2023-072943)
Supplement: online supplemental file 2 [file bmjopen-14-7-s002.pdf]

Table 2. Distribution of health conditions disclosed (expressed as percent of the number of clients who had a job start) by client group.

	JSA <50	JSA 50+	ESA <50	ESA 50+
Addictions	1.2	1.2	12.8	8.8
Anxiety	2.2	2.5	36.5	28.6
Cardiovascular	1.0	5.8	3.8	16.5
Depression	4.3	6.6	47.3	50.0
Diabetes	1.2	5.2	3.4	4.9
Fibromyalgia/CFS	0.1	0.1	0.9	1.1
Gastrointestinal	0.5	0.9	5.4	7.7
Learning disability	1.0	0.3	1.0	0.0
Musculoskeletal	5.2	20.9	34.9	60.4
Neurological	1.1	1.7	7.6	4.4
No health conditions	81.3	58.6	4.8	3.3
Other physical	1.2	3.6	5.0	4.4
Prefer not to say	0.7	2.2	6.3	4.9
Respiratory	3.6	5.8	5.5	7.7
Sensory conditions	0.5	1.7	1.8	3.3
Severe mental health	0.4	0.9	6.9	3.8
Unknown	0.1	0.0	0.6	0.0
Total with any health condition	*18.7*	*41.4*	*95.2*	*96.7*
