# Supplementary material for: Investigating sustainability in work after participating in a welfare-to-work initiative using a 2-year cohort study of Work Programme participants in Scotland
Source: BMJ Open. 2024 Jul 3;14(7):e072943. doi: 10.1136/bmjopen-2023-072943 (PMC11733907; doi:10.1136/bmjopen-2023-072943)
Supplement: online supplemental file 3 [file bmjopen-14-7-s003.pdf]

Table 3. Percent part-time/full-time jobs by benefit type & age group [*may not include this table*]

Client group	Part-time jobs, %	Full-time jobs, %	Unknown jobs, %
JSA under 50	32.6	62.5	4.9
JSA over 50	32.6	63.2	4.2
ESA under 50	46.0	51.5	2.6
ESA over 50	56.5	41.8	1.7

Full-time work was defined as 30 hours +, part-time work was defined as 29 hours and less.

*Test of association on part-time/full-time job and benefit type/age group $p < 0.001$
