# Supplementary material for: Investigating sustainability in work after participating in a welfare-to-work initiative using a 2-year cohort study of Work Programme participants in Scotland
Source: BMJ Open. 2024 Jul 3;14(7):e072943. doi: 10.1136/bmjopen-2023-072943 (PMC11733907; doi:10.1136/bmjopen-2023-072943)
Supplement: online supplemental file 4 [file bmjopen-14-7-s004.pdf]

Table 4. Distribution of number of jobs started by benefit type and age group (excluding those with none).

	Client group			
Number of job starts	JSA under 50 Number (%)	JSA over 50 Number (%)	ESA under 50 Number (%)	ESA over 50 Number (%)
1	2805 (57%)	425 (61%)	489 (71%)	138 (76%)
2	1261 (26%)	185 (27%)	130 (19%)	38 (21%)
3	542 (11%)	54 (8%)	50 (7%)	6 (3%)
4	198 (4%)	19 (3%)	12 (2%)	0 (0%)
5	61 (1%)	5 (1%)	3 (0.4%)	0 (0%)
6	26 (0.5%)	2 (0.3%)	0 (0%)	0 (0%)
7	18 (0.4%)	1 (0.1%)	0 (0%)	0 (0%)
8	4 (0.1%)	2 (0.3%)	1 (0.1%)	0 (0%)
9	3 (0.1%)	0 (0%)	0 (0%)	0 (0%)
10	1 (0.0%)	0 (0%)	0 (0%)	0 (0%)
Total number of clients	*4919*	*693*	*685*	*182*
Total number of jobs	*8401*	*1093*	*970*	*232*
