# Supplementary material for: Investigating sustainability in work after participating in a welfare-to-work initiative using a 2-year cohort study of Work Programme participants in Scotland
Source: BMJ Open. 2024 Jul 3;14(7):e072943. doi: 10.1136/bmjopen-2023-072943 (PMC11733907; doi:10.1136/bmjopen-2023-072943)
Supplement: online supplemental file 5 [file bmjopen-14-7-s005.pdf]

Table 5. *Time in Employment Index* (proportion of total time on Work Programme spent in employment) by benefit type and age group.

Client group	Number of clients	Number of clients with a job start	% of clients with job start	Total number of jobs started	Mean number of jobs started
JSA under 50	7590	4919	65%	8401	1.71
JSA over 50	1406	693	49%	1092	1.58
ESA under 50	3000	685	23%	970	1.42
ESA over 50	1322	182	14%	232	1.27
All clients	*13318*	*6479*	*49%*	*10696*	*1.65*
