# Supplementary material for: Investigating sustainability in work after participating in a welfare-to-work initiative using a 2-year cohort study of Work Programme participants in Scotland
Source: BMJ Open. 2024 Jul 3;14(7):e072943. doi: 10.1136/bmjopen-2023-072943 (PMC11733907; doi:10.1136/bmjopen-2023-072943)
Supplement: Supplementary file 6 [file bmjopen-2023-072943.reviewer_comments.pdf]

PEER REVIEW HISTORY

BMJ Open publishes all reviews undertaken for accepted manuscripts. Reviewers are asked to complete a checklist review form (**http://bmjopen.bmj.com/site/about/resources/checklist.pdf**) and are provided with free text boxes to elaborate on their assessment. These free text comments are reproduced below.

This paper was submitted to a another journal from BMJ but declined for publication following peer review. The authors addressed the reviewers' comments and submitted the revised paper to BMJ Open. The paper was subsequently accepted for publication at BMJ Open.

ARTICLE DETAILS

**TITLE (PROVISIONAL)**	Investigating sustainability in work after participating in a welfare to work initiative using a 2-year cohort study of Work Programme participants in Scotland.
**AUTHORS**	Brown, Judith; Walker, Simon; McQuaid, Ronald; Katikireddi, Srinivasa; Leyland, Alastair; Frank, John; Mackay, Daniel; Macdonald, Ewan

VERSION 1 – REVIEW

**REVIEWER**	Sealey, Clive<br>University of Worcester, Allied Health and Community
**REVIEW RETURNED**	20-Jun-2023

**GENERAL COMMENTS**	The analysis in the paper does seem to suggest that ESA clients sustain their employment better than healthier unemployed clients, which is something that would be a positive in relation to the Work Programme. This would also be especially relevant in relation to the changing demographics of the population, and the concomitant change in health profile that this is very likely to result in. The sequence analysis graphs do provide a good visual representation of the differences between the cohorts, especially in relation to the ESA clines over 50. The data for those who left the programmes does indicate that clients on ESA tend to take longer to find a job and so stay on the programme longer than JSA clients, which is perhaps not surprising bearing in mind their generally worse health, but is nonetheless a relevant point to note.<br><br>However, there are a number of issues that need to be addressed in the paper:<br><br>Definition of sustainability in work - this needs to be more clearly defined in the article, so that it is clear at which point an employment is sustainable. Is there a specific cut-off point i.e. 6 months, or is this simply a reference to the criteria set out for the Work Programme? This is especially relevant in the context of Table 2, which shows both JSA groups having higher mean and median time in employment than ESA groups, which relates back again to how sustainability is defined in the study.<br><br>The paper would benefit from a more detailed breakdown of the age profile of participants. The categories of under 50 and over 50

	are too broad on their own. It may be the case that the results were skewed by the the fact that the age profile of one cohort was different from another. For example, if the age profile for the category JSA under 50 was predominantly under 35, this would be significant in affecting the finding. The age profile needs to be more detailed, as a way to understand the categories used.<br><br>Similarly, a gender profile cross tabulation of participants would also be useful, as a way to ensure that the results are also not skewed. For example, it is well known that women are more likely to be classified as disabled than men, and therefore if the gender profile of participants is skewed towards a particular gender, this could have a bearing on the findings.<br><br>The paper could be clearer on the 17 categories that they recoded the health conditions into, and what was the basis for this.<br><br>The definitions of part time and full time work used in the paper are not the usual definitions, especially full time. The basis for these definitions needs to be made clearer.<br><br>The paper only briefly considers the possibility that the findings for under 50 JSA in relation to higher mean number of jobs is due to the nature of the this cohort itself i.e. younger people tend to have less stable career histories than older people. This needs to be discussed as a possible confounding factor.<br><br>On page 16, the paper briefly mentions that ESA clients could not leave the Work Programme early due to the requirement for longer job outcomes and sustainment payments for contractors. These are not discussed in any detail in terms of their possible significance of the findings, as they suggest that the Programme was geared towards enabling ESA clients to stay longer in work, more for so than for JSA clients. This needs to be discussed in more detail.<br><br>These issues limit the claims made in the paper. In particular, the claim that ESA over 50s go into more sustainable work is not wholly supported by the evidence. Addressing the issues highlighted above would facilitate the robustness of the claims made in the paper.

**REVIEWER**	von Fintel, Dieter<br>Stellenbosch University, Department of Economics
**REVIEW RETURNED**	23-Jun-2023

**GENERAL COMMENTS**	This paper examines transitions back into the workplace of two groups of welfare-to-work recipients: one group of recipients has disabilities and the other is a group of unemployment claimants.<br><br>The abstract briefly refers to "multimorbidity" as a health outcome. Apart from comparing recipients with disabilities to those without disabilities, the abstract does not make it very clear how this paper is about a topic in "health". To fit into this journal, this needs to be stipulated more clearly and explicitly.<br><br>To motivate the study, the introduction cites many statistics relevant to the entire United Kingdom. In the methods section, however, it becomes clear that the study area is limited only to Scotland. For the benefit of the reader and to support the

	relevance of the study, the authors need to motivate that the labour market situation of chronically ill individuals and the long-term unemployed is similar or worse in Scotland to the entire United Kingdom. If Scotland is different to the rest of the UK, then that context must be spelled out more clearly in the introduction. It is possible that the situation is not as severe in Scotland, which potentially reduces the relevance of this particular study?<br><br>The appendix incorrectly labels Table 1 as Figure 1. Figure 1 is not shown in the manuscript I was given.<br><br>Figure 2 presents group-specific sequence analysis curves. The authors are concerned by presenting "too much information" and choose 100 randomly sampled individuals from each client group. The more appropriate way to present this information is by using survivor curves or cumulative hazard functions. This would also make it easier to compare the hazard rates (representing job entry) across the 4 groups on one set of axes. I think that the sequence graphs are useful for illustrative purposes, but comparative hazard functions are the more appropriate to present the full picture (with a full sample) and to aid effective comparisons across groups. The hazard curves can also account for multiple events and data censoring. Please update this.<br><br><br>Table 1: My statistical intuition suggests that the proportions are statistically significant across the client groups. Please report the significance of the differences between age groups (within programme) and the significance of differences between programmes (within age groups).<br><br>The analysis of health conditions is not reported in any tables. However, comparisons across client groups are reported in text. While there are differences in health outcomes across client groups, no attempt is made to show whether these patterns are related to or overlap with differences in employment trajectories across groups. Of course, because the data are observational, it is not possible to determine whether the health conditions have an impact on employment or whether employment has an impact on the health outcomes. But at least reporting the associations (by estimating a survivial time regression model) would help us to understand how programme type, *initial* health conditions at the start of the programme and age relate to employment spells and transitions. A model would also help to understand which factors remain significant when all are included in a model simultaneously - in other words, does initial health mediate/moderate the effects of age and programme? Not reporting these relationships strongly detracts from this being a paper about "health". Rather, the paper is about employment of different groups. The authors demonstrate limited links to client health conditions in indirect and limited ways.<br><br>Are ESA workers staying in jobs longer because the type of jobs they enter are specifically picked for them to suit their abilities? My question is whether the ESA provides additional support that the JSA does not - this is briefly referred to in the discussion. The potential result of the (longer and more intensive) support is a better *match* between work seekers' attributes and the requirements of employers/vacancies. They may be staying longer in these jobs because of higher quality matches - though see my comments about censoring below. Similarly, *older* ESA clients

	may also be getting better support that prevents them from immediately entering a job, but allowing them to find jobs that are better suited to them over the long-term. I think more needs to be said about the support provided by the programmes to be able to interpret these results effectively.<br><br>"This study has demonstrated the importance of multimorbidity" is not a correct claim. Because the actual morbidities have not been correlated with employment outcomes, this is a very tenuous claim.<br><br>It is only in the limitations section that we are told that ESA participants were unlikely to attrite, while many JSA participants left the programme early. While the limitations section is the appropriate place for such a statement, I believe the reader should be made aware of this much earlier - perhaps in the methods section of the manuscript. One of the major findings of this paper (job duration of ESA workers > JSA workers) is strongly influenced by this *major* limitation. The reader is led to believe that this is a robust finding, and only late in the manuscript finds out that it is not reliable. Attempts to compare ESA clients to non-attriting JSA samples does not solve the problem, because these are not *representative* of that group. The only way I can think that this problem can start to be solved is using a survival time model that accounts for right-censoring, and which can statistically assess the differences on the *entire* sample. I am not convinced by the findings from the current analysis.

VERSION 1 – AUTHOR RESPONSE

Reviewer: 1
Dr. Clive Sealey, University of Worcester

Comments to the Author:

1. The analysis in the paper does seem to suggest that ESA clients sustain their employment better than healthier unemployed clients, which is something that would be a positive in relation to the Work Programme. This would also be especially relevant in relation to the changing demographics of the population, and the concomitant change in health profile that this is very likely to result in. The sequence analysis graphs do provide a good visual representation of the differences between the cohorts, especially in relation to the ESA clines over 50. The data for those who left the programmes does indicate that clients on ESA tend to take longer to find a job and so stay on the programme longer than JSA clients, which is perhaps not surprising bearing in mind their generally worse health but is nonetheless a relevant point to note.
a. Thank you, we have made this clearer within the text.

However, there are a number of issues that need to be addressed in the paper:

2. Definition of sustainability in work - this needs to be more clearly defined in the article, so that it is clear at which point an employment is sustainable. Is there a specific cut-off point i.e. 6 months, or is this simply a reference to the criteria set out for the Work Programme? This is especially relevant in the context of Table 2, which shows both JSA groups having higher mean and median time in employment than ESA groups, which relates back again to how sustainability is defined in the study.
a. Thank you for asking us to clarify this issue. We have included a more specific definition of the term sustainability in work. Sustainability for the purposes of our work was defined by individuals remaining

in work for the duration of the programme. Crucially, the Scottish Work Programme was uniquely focused on the sustaining (i.e. the retaining) of individuals within work. Renumeration processes were justified by continued retention. Providers claimed a job outcome payment after a client had moved into and been in employment for three or six months, depending on how far they were from the labour market (i.e. which payment group they were in). After receiving an initial job outcome payment, providers claimed sustainment payments for every four weeks a client remained in employment, again depending on the client's payment group (either 13, 20 or, for those furthest from the labour market, 26 four-weekly payments). The payments were not for a particular job but for accumulated employment, irrespective of how many employers or jobs this time in employment was made up of. This has been made clearer within the text:
b. This is stated on 10-11. Additionally, a 6WRI (Six-Month Work Retention Index) was developed to identify if the censoring of the data influenced the results recorded (i.e. some ESA clients started work near the end of their two years on the WP and we did not have client data after that time). This index therefore only considered employment during the six months after first starting a job and excluded all those who first started a job in the last 6 months of the Work Programme (n=614).
The paper would benefit from a more detailed breakdown of the age profile of participants. The categories of under 50 and over 50 are too broad on their own. It may be the case that the results were skewed by the the fact that the age profile of one cohort was different from another. For example, if the age profile for the category JSA under 50 was predominantly under 35, this would be significant in affecting the finding. The age profile needs to be more detailed, as a way to understand the categories used.
c. This is a very good point. Our response to this question is to refer back to the overall conclusion of the research: to establish that individuals over 50 face significant issues in retaining or returning to work. This is to not say that further research is not required to focus on the differences between age groups, however, it is evident for the purposes of this research that the split at 50 has significant consequences for individuals. This is has been evidenced and outlined by the ONS (2022), most recently in association with the COVID epidemic.
i. Office for National Statistics (ONS), released 27 September 2022, ONS website, statistical article, “Reasons for workers aged over 50 years leaving employment since the start of the coronavirus pandemic: wave 2”
d. The ONS use ‘Under 50 / 50 Plus’ as standard delineators. For this reason, we have too.
e. The purpose of this analysis is descriptive and we are therefore interested in reporting the employment outcomes of different population groups (stratified by benefit receipt and age). We are therefore not seeking to assess if the differences are due to benefit status (or indeed due to age). Rather, our interest is in quantifying differences in employment outcomes across specific subgroups that are the subject of policy targeting. For this reason, age is not a confounder but rather a part of the definition of the exposure. We do agree that exploration of other age cut-offs would be informative, but unfortunately we no longer have access to these deidentified administrative data.
f. This has been outlined on p.15

3. Similarly, a gender profile cross tabulation of participants would also be useful, as a way to ensure that the results are also not skewed. For example, it is well known that women are more likely to be classified as disabled than men, and therefore if the gender profile of participants is skewed towards a particular gender, this could have a bearing on the findings.
a. This is an excellent point. In response we would point to the focus of the paper and the need for explicit focus on age over other factors. However, the impact of Gender is an important one and we agree should be clearer within the text. As a result, we have included more detail about the results categorised by age and gender demonstrating a strong negative relationship between age and having a job start for both JSA and ESA clients. WeWe now note that the gender profile is an important factor and is suitable for future follow up, however due to the constraints of this paper and the points to be made that are fundamental to it, gender is not identified as a priority focus for this study, but

recognised as a contributing factor We do agree that exploration of gender would be informative, however, this is a descriptive analysis and unfortunately we no longer have access to these deidentified administrative data..

4. The paper could be clearer on the 17 categories that they recoded the health conditions into, and what was the basis for this.
a. Thank you for this point – this has been made clearer in the text and a table has been included for clarity also.
INCLUDE QUOTE TOO.

5. The definitions of part time and full time work used in the paper are not the usual definitions, especially full time. The basis for these definitions needs to be made clearer.
a. This is a good point which has been clarified in the text. In response we would like to make the point that classifying full time work as 30+ hours reflect government policy relevant to employment (for instance at least 30 hours is the limit for Working Tax Credit) (https://www.gov.uk/working-tax-credit) and variable work patterns. The Labour Force Survey (LFS) uses respondents' self-defined full time work classification with a median of around 34 hours per week for women working full time over the study period, so if 35+ hours was taken as full-time work then over half of these women would be excluded. (Source: ONS 'HOUR01 SA: Actual weekly hours worked (seasonally adjusted)' Accessed 30/8/23
https://www.ons.gov.uk/employmentandlabourmarket/peopleinwork/earningsandworkinghours/datasets/actualweeklyhoursworkedseasonallyadjustedhour01sa
b. Please view Table 3 on page 28,
c.

6. The paper only briefly considers the possibility that the findings for under 50 JSA in relation to higher mean number of jobs is due to the nature of the this cohort itself i.e. younger people tend to have less stable career histories than older people. This needs to be discussed as a possible confounding factor.
a. Thank you for raising this. This is another important point that needed to be more clearly addressed. The limitation section of the paper has been expanded to include a response to this.

7. On page 16, the paper briefly mentions that ESA clients could not leave the Work Programme early due to the requirement for longer job outcomes and sustainment payments for contractors. These are not discussed in any detail in terms of their possible significance of the findings, as they suggest that the Programme was geared towards enabling ESA clients to stay longer in work, more for so than for JSA clients. This needs to be discussed in more detail.
a. Thank you for raising this. This has been raised as a limitation – with the following response - While the availability of such rich data on unemployment to employment transitions is a major strength of this study there are also some limitations to the data. Once Ingeus received all eligible payments for a client no further data on that client was available. Hence, we created the 'left programme' status for these 1187 clients. Although these clients may have remained in employment due to their good work history for the remaining two years of the Work Programme, we could not assume this, so we censored follow-up for subjects who 'left the programme,' in our calculation of the time in employment index and work retention index. These clients were generally JSA clients (due to payment rules mainly Payment Groups one and two, (26)) who had found work quickly and sustained in the same job. They will have had to work for at least 550 days for Ingeus to collect all payments (and record their data). The only clients who could not leave the Work Programme early were ESA clients from payment groups seven and nine because of the different payment structure with much longer job outcome and sustainment payments that providers would collect for these clients.

8. These issues limit the claims made in the paper. In particular, the claim that ESA over 50s go into more sustainable work is not wholly supported by the evidence. Addressing the issues highlighted above would facilitate the robustness of the claims made in the paper.
a. Thank you for raising this, this is an interesting point. We have attempted to make it clearer within the text that the statistical evidence demonstrates that those over 50 typically returned and remained in the work place when compared to their younger counterparts. The reason for this have been questioned within the paper.
b. As above, the results are descriptive and therefore the finding of entering sustainable work among ESA recipients aged over 50 years we believe remains valid. While we agree that differences in age might help explain why work is sustained among ESA recipients (relative to JSA recipients), these are part of the differences between the exposure groups, rather than a confounding factor. Given that long-term conditions are very common among older working age people, and relatively uncommon in early working age, we believe our descriptive approach of reporting the outcomes actually experienced is a reasonable one (although alternative approaches to address other questions could have been pursued).

Reviewer: 2
Dr. Dieter von Fintel, Stellenbosch University

Comments to the Author:
This paper examines transitions back into the workplace of two groups of welfare-to-work recipients: one group of recipients has disabilities and the other is a group of unemployment claimants.

1. The abstract briefly refers to "multimorbidity" as a health outcome. Apart from comparing recipients with disabilities to those without disabilities, the abstract does not make it very clear how this paper is about a topic in "health". To fit into this journal, this needs to be stipulated more clearly and explicitly.
i. Thank for raising this really good point. Multi-morbidity is a key factor within this study as part of the research. We have improved within the introduction and the discussion section the consideration of multi-morbidity.

2. To motivate the study, the introduction cites many statistics relevant to the entire United Kingdom. In the methods section, however, it becomes clear that the study area is limited only to Scotland. For the benefit of the reader and to support the relevance of the study, we need to motivate that the labour market situation of chronically ill individuals and the long-term unemployed is similar or worse in Scotland to the entire United Kingdom. If Scotland is different to the rest of the UK, then that context must be spelled out more clearly in the introduction. It is possible that the situation is not as severe in Scotland, which potentially reduces the relevance of this particular study?
i. Thank for raising this. This has occurred because of the editing process from the larger paper to the current form. To make this viable we have returned some of the information on Scotland. More widely, a lot of the information is typically focused on the UK over Scotland – the data presented is designed to provide more information on the impact of this programme on Scotland and demonstrate the issues faced by Older workers.
3. The appendix incorrectly labels Table 1 as Figure 1. Figure 1 is not shown in the manuscript I was given.
a. Thank you for bringing this up. This has been amended and all of the tables have been properly labelled.
4. Figure 2 presents group-specific sequence analysis curves. We are concerned by presenting "too much information" and choose 100 randomly sampled individuals from each client group. The more appropriate way to present this information is by using survivor curves or cumulative hazard functions. This would also make it easier to compare the hazard rates (representing job entry) across the 4 groups on one set of axes. I think that the sequence graphs are useful for illustrative purposes, but

comparative hazard functions are the more appropriate to present the full picture (with a full sample) and to aid effective comparisons across groups. The hazard curves can also account for multiple events and data censoring. Please update this.
a. Sequence analysis curves have a different purpose to cumulative hazard functions. They are intended to illustrate clusters and their frequency among a population. As such, they are for visual purposes only. The statistical analysis, including those which the conclusions are based on, use the whole analytical sample. Kaplan-Meier curves cannot easily be used for illustrating multiple events although of course Cox regression (and other survival analyses) can account for them. More importantly, they do not illustrate the clustering of events within individuals in the way that sequence analysis does. For this reason, we believe that sequence analysis is a useful visual tool for illustrating the data, but would like to reassure the reviewer that the entire analytical dataset (rather than a random sample) has been used for calculating summary statistics.

Table 1: My statistical intuition suggests that the proportions are statistically significant across the client groups. Please report the significance of the differences between age groups (within programme) and the significance of differences between programmes (within age groups).
a. The analysis of health conditions is not reported in any tables. However, comparisons across client groups are reported in text. While there are differences in health outcomes across client groups, no attempt is made to show whether these patterns are related to or overlap with differences in employment trajectories across groups. Of course, because the data are observational, it is not possible to determine whether the health conditions have an impact on employment or whether employment has an impact on the health outcomes. But at least reporting the associations (by estimating a survivial time regression model) would help us to understand how programme type, *initial* health conditions at the start of the programme and age relate to employment spells and transitions. A model would also help to understand which factors remain significant when all are included in a model simultaneously - in other words, does initial health mediate/moderate the effects of age and programme? Not reporting these relationships strongly detracts from this being a paper about "health". Rather, the paper is about employment of different groups. We demonstrate limited links to client health conditions in indirect and limited ways.Ultimately, we are describing employment outcomes in relation to population subgroups, but our intention is not to explore the role of specific factors.

Are ESA workers staying in jobs longer because the type of jobs they enter are specifically picked for them to suit their abilities? My question is whether the ESA provides additional support that the JSA does not - this is briefly referred to in the discussion. The potential result of the (longer and more intensive) support is a better *match* between work seekers' attributes and the requirements of employers/vacancies. They may be staying longer in these jobs because of higher quality matches - though see my comments about censoring below. Similarly, *older* ESA clients may also be getting better support that prevents them from immediately entering a job, but allowing them to find jobs that are better suited to them over the long-term. I think more needs to be said about the support provided by the programmes to be able to interpret these results effectively.

It is only in the limitations section that we are told that ESA participants were unlikely to attrite, while many JSA participants left the programme early. While the limitations section is the appropriate place for such a statement, I believe the reader should be made aware of this much earlier - perhaps in the methods section of the manuscript. One of the major findings of this paper (job duration of ESA workers > JSA workers) is strongly influenced by this *major* limitation. The reader is led to believe that this is a robust finding, and only late in the manuscript finds out that it is not reliable. Attempts to compare ESA clients to non-attriting JSA samples does not solve the problem, because these are not *representative* of that group. The only way I can think that this problem can start to be solved is

using a survival time model that accounts for right-censoring, and which can statistically assess the differences on the *entire* sample. I am not convinced by the findings from the current analysis.
a. Thank for raising this. This has occurred because of the editing process from the larger paper to the current form. Changes have been made to make this clearer in the introduction and the discussion. We would like to clarify that our analytical approach is based on survival time (proportion of time employed) and therefore it accounts for right-censoring in the same way as a survival time model. The entire sample is used in the analysis, but some of the person-time is right-censored. We note and discuss the implications of this potential bias, as described in our response to xxx

VERSION 2 – REVIEW

**REVIEWER**	Sealey, Clive<br>University of Worcester, Allied Health and Community
**REVIEW RETURNED**	12-Dec-2023

**GENERAL COMMENTS**	The revisions provide a more nuanced paper than the original submission, as the data and findings are more aligned with each other. This makes the paper clearer and read much better than previous.<br><br>In the light of the revisions, the Conclusion section of the Abstract may need to be revised. It is clear that a major finding of the paper now is that the' human capital approach' is imprtant to the sustainability of employment for individuals on simialr schemes, and this needs to be emphasised in the Abstract. This is also something that could be discussed in more detail in the Conclusion, as there is a growing body of data that supports this, and this paper could provide an important consideration of this.

VERSION 2 – AUTHOR RESPONSE

Dr. Clive Sealey, University of Worcester
Comments to the Author:
The revisions provide a more nuanced paper than the original submission, as the data and findings are more aligned with each other. This makes the paper clearer and read much better than previous.
• Thank you kindly for taking the time to reassess and review the paper again and give excellent feedback.

In the light of the revisions, the Conclusion section of the Abstract may need to be revised. A major finding of the paper now is that the' human capital approach' is imprtant to the sustainability of employment for individuals on simialr schemes, and this needs to be emphasised in the Abstract. This is also something that could be discussed in more detail in the Conclusion, as there is a growing body of data that supports this, and this paper could provide an important consideration of this.
• Thank you for this point. As part of the suggested changes the abstract has been rewritten, and these points have been included in the changes.

Reviewer: 1
Competing interests of Reviewer: None
